# Attitudes and practices on antibiotic use and its emerging threats among Lebanese dairy veterinarians: a case study from a developing country

**DOI:** 10.3389/fvets.2023.1284656

**Published:** 2023-11-30

**Authors:** Iman Dankar, Hussein F. Hassan, Mireille Serhan

**Affiliations:** ^1^Department of Nutritional Sciences, Faculty of Health Sciences, University of Balamand, Beirut, Al Koura, Lebanon; ^2^Nutrition Program, Department of Natural Sciences, School of Arts and Sciences, Lebanese American University, Beirut, Lebanon

**Keywords:** dairy, farms, antimicrobial resistance, Lebanon, veterinarians

## Abstract

**Introduction:**

This study aimed to explore how veterinarians (vets) rationalized their prescribing decisions for antimicrobial (AM) uses, the barriers they perceived to implement proper farm management in Lebanon, and the consecutive threats that might arise concerning the emergent spread of antimicrobial resistance in animals and the population.

**Methods:**

Amid the COVID-19 pandemic, phone call interviews were conducted with 34 veterinarians working in different demographic regions across the country. Data were analyzed qualitatively using an inductive thematic analysis.

**Results and discussion:**

The majority of veterinarians called for responsible antibiotic use. The prescribing decision of veterinarians was based mainly on suspected disease from field examination, farmer’s reports via phone calls, and the ability of the farmer to cover antibiotic costs. Very few veterinarians referred to laboratory diagnosis before prescribing a specific AM due to many obstacles. This study uncovered the absence of a trust relationship between veterinarians and farmers in Lebanon. Veterinarians provided different insights into farming practices, reflecting that farmers, in general, lack proper knowledge and implementation of farm management and that they mainly treat the herd on their own, especially in light of the current unprecedented economic crisis that Lebanon has been facing in the last three years. Above all, veterinarians revealed that AM resistance in Lebanon is markedly spreading, which calls for a serious and instantaneous set of governmental policies and regulations.

## Introduction

Antibiotics are utilized in the dairy sector for both therapeutic and control purposes. These have been shown to offer several advantages, including disease treatment, increased productivity, and a reduction in food-borne diseases, caused by pathogens, such as pathogenic *E. coli*, *S. aureus*, *S. pneumonia*, *Actinobacillus pleuropneumoniae*, mycoplasma, *Vibrio*, and others (1, 2). The World Organisation for Animal Health (WOAH) has mentioned that the use of antimicrobial drugs has advanced global public health, animal health, and food safety and security (3). They can, however, cause a variety of complications, such as the rise of antibiotic-resistant bacteria (4). The global concern over antimicrobial resistance (AMR) is increasing, with human deaths worldwide from AMR predicted to rise from 700,000 to 10 million by the year 2050 if action is not taken to address the problem (5). The inappropriate use of antibiotics defined by the World Health Organization (WHO) as over-prescription, under-prescription, inappropriate dosing, incorrect duration of treatment, or the incorrect choice of drug for the relevant organism, can exacerbate these problems (6). This debate has led to regulations concerning decreasing antimicrobial uses (AMU) and regulating AMU in animal husbandry at the level of dairy farmers and veterinary practices with specific targets for each (7). Moreover, according to WOAH (8), monitoring the implementation of standards related to AMU and resistance requires building and analyzing indicators using various data sources, such as qualitative and quantitative research.

Currently, in most developing countries, the majority of antimicrobials in the dairy sector are applied by the farmer in an uncontrolled ambiguous way, consequently increasing the risk of AMR among animals and humans (9–13). Both farmers and veterinarians share responsibility for the prudent use of antimicrobials (AM) on the farm level. It has been reported that veterinarian-farmer relationships influence the AMU in livestock, such that positive well-established relationships aid in antibiotic stewardship and restricted AMU. The farmer’s trust was crucial for implementing a new treatment strategy for *E. coli* mastitis in Sweden (14), which meant that instead of quinolones, frequent milking, and anti-inflammatory drugs were used. Similarly, Green et al. (15) reported that developing sustainable dairy farms in the United States, ensuring that healthy management is achieved without relying on antibiotics is attributed to the trusted advisory relationship with veterinarians. On the contrary, veterinarians in South Peru and Jordan found that there is little to do to improve antibiotic practices among their clients who prefer to use AM on their own both due to a lack of education and trust relationship with veterinarians (16, 17). A study conducted in Turkey highlights two significant knowledge gaps in this regard; (1) understanding farmer and veterinary prescription and use, and (2) the need for comparable data on prescription and use (11).

In Lebanon, veterinarians obtain their degrees in veterinary medicine after 6 years of full-time study at a university, they are then organized into a veterinarian’s syndicate under the Ministry of Agriculture. The Ministry of Agriculture in coordination with the veterinarians’ syndicate association informs the veterinarians about the new laws and guidelines concerning antibiotic use and dictates the permissible kinds of antibiotics to be imported into the country according to the World Health Organization recommendations. Though, until now no specific studies to reflect the implementation of those practices in Lebanon have been performed. Dankar et al. (9) conducted a first of its kind study in Lebanon about dairy farmers’ attitudes towards AMU and AMR. Yet, until now, data about AMU among dairy veterinarians in Lebanon has been unavailable. However, a thorough knowledge of AMU by dairy veterinarians and its related factors is required to support the efforts done in controlling the emergence of AMR, and a necessary step for designing, implementing, and evaluating regional and local interventions directed at optimizing the use of veterinary drugs and improving farming practices.

In contrast to quantitative questionnaires with closed-ended questions, the qualitative methodological approach allows one to consider a wide range of topics and to spot unforeseen replies, for instance, analyzing veterinarian practices, comparing and contrasting them with the farmers’ practices while understanding relying on sociological and economical motives (9).

Antibiotic residues in dairy products in Lebanon were tackled from the food processing angle (18, 19). The aim of our first of its kind research in Lebanon was to (1) address how veterinarians with different work experiences and backgrounds rationalize decisions for prescribing antibiotics and AMU, (2) to explore veterinarians’ attitudes and perceptions towards farming management and AMR, and (3) to assess challenges related to farm management and antimicrobial prescribing practices.

## Materials and methods

### Study design

A semi-structured interview focusing on antibiotic prescriptions and use in dairy farms from the veterinarian’s perspective was carried out in Lebanon, and conducted with 34 veterinarians across the country. In particular, interviews focused precisely on the knowledge about the choice of antibiotics, along with practices and the possible influence of external factors affecting antibiotic or alternative medicine used by Lebanese veterinarians. The exploratory study was intended to give unique insights into a mostly neglected issue in Lebanon.

### Selection of veterinarians

The Lebanese Order of Veterinarians is the veterinarian syndicate organization that is held under the Ministry of Agriculture and has a total of 55 registered and accredited dairy farm veterinarians’ members. Contact information was obtained from the order. Textphone messages, which contain brief descriptions of the study, the aim, and the background of the investigators were sent to the 55 veterinarians, from whom 34 agreed to be part of the study. More descriptive information for veterinarians interviewed is available in Table 1. Interviews with the veterinarians were conducted over the phone in May and June 2021, and each lasted 80 to 120 min.

**Table 1:** Summarizing the participating dairy vets demographics, regarding age, years of experience, education qualification, and state of work per governorate region.

Dairy vets (V)	Age (years)	Years of experience	Post graduate qualifications	State of work	Working region per governorate
V1	29	6	Master’s degree	Private	Mount Lebanon
V2	46	20	Master’s degree	Private	Nabatiye
V3	28	5	Master’s degree	Private	Baalbeck-Hermel
V4	54	26	PhD	Private	Mount Lebanon
V5	32	5	Master’s degree	Private	Bekaa-Zahle
V6	52	15	PhD	Private	North Lebanon/South Lebanon/Bekaa-Zahle/Mount Lebanon/Beirut (Strolling Vet)
V7	53	25	Master’s degree	Public*	Nabatiye
V8	38	8	Master’s degree	Private	Mount Lebanon
V9	54	28	PhD	Private	All governorates (Strolling vet)
V10	34	10	Master’s degree	Private	All governorates (Strolling Vet)
V11	34	9	Master’s degree	Private	North Lebanon
V12	35	5	Master’s degree	Private	North Lebanon/South Lebanon
V13	30	4	Master’s degree	Public	Mount Lebanon
V14	31	5	Master’s degree	Private	Bekaa-Zahle
V15	33	7	Master’s degree	Private	Bekaa-Zahle
V16	69	39	PhD	Private	North Lebanon/Nabatiye/Beirut (Strolling Vet)
V17	70	40	Master’s degree	Public	South Lebanon
V18	54	25	Master’s degree	Private	Nabatiye
V19	32	6	Master’s degree	Public	Bekaa-Zahle
V20	40	18	Master’s degree	Private	Akkar/North Lebanon/Nabatiye/Beirut (Strolling Vet)
V21	54	26	Master’s degree	Private	Nabatiye
V22	52	20	Master’s degree	Public	North Lebanon
V23	48	20	Master’s degree	Public	Akkar
V24	43	13	Master’s degree	Private	Mount Lebanon
V25	55	26	Master’s degree	Private	South Lebanon
V26	30	6	Master’s degree	Private	Mount Lebanon
V27	33	7	Master’s degree	Private	North Lebanon/Bekaa-Zahle
V28	32	7	Master’s degree	Private	Bekaa-Zahle
V29	36	5	Master’s degree	Private	Beirut
V30	32	5	Master’s degree	Private	Mount Lebanon
V31	42	8	Master’s degree	Public	North Lebanon
V32	68	35	Master’s degree	Private	Mount Lebanon
V33	49	19	Master’s degree	Public	Baalbeck-Hermel
V34	35	11	Master’s degree	Private	Beirut

*Public with Governmental Agricultural Ministry.

### Interview guide

Findings and survey questionnaires from other studies regarding antibiotic uses and practices by farmers and veterinarians were considered as references for developing the interview guide questions (20–22).

The interview guide was a flexible platform that supports open follow-up questions and reminders. The interviewer underwent a training course on the tips and qualifications of qualitative research to enhance the effectiveness of the study and avoid any bias while reporting data. Accordingly, interviewers were instructed to use neutral and non-leading language. Questions were framed in a way that encouraged open responses from participants, rather than insinuating particular answers. Interviewees were left to express themselves on issues that they found relevant to the subject. Sometimes, they were provided with choices to direct the focus of the question and prevent any deviation from the topic.

The interview guide was structured into three main themes, with subthemes within this as follows:

(1) Veterinarians’ practices towards AMU on dairy cattle

– *Veterinarians’ approach towards successive AM treatment failure*
– *Factors that govern veterinarians’ AM prescription decisions*
– *Conditions that direct veterinarians’ use of AM as a preventive measure*
– *Veterinarians’ attitude towards AM misuse*
– *Willingness of veterinarians to reduce AMU on Farm*

(2) Farmer-veterinarian relationship.
(3) Attitudes of veterinarians toward AMR and its scale threat in Lebanon.

### Pilot testing

Interview questions were first pretested on 5 veterinarians, to identify any potential issues related to bias and make necessary adjustments to the questions and interview technique in the upcoming interviews. All interviews were held via phone calls due to the COVID-19 pandemic in place. The length of each exchange (80–120 min) encourages discussion. Semi-structured interviews were held by the first author (ID), based on the interview guide developed with input from all authors.

Questions were designed in English and verbally translated into the local Arabic language during the inset of questioning, as some of the vets felt it easier to relate all their information in Arabic. Recorded answers were then translated back into English. While translating, we made sure to maintain confidentiality of the results, the recordings and answers were transcribed by the first author in a way to retain the tone and the meaning of the responses while keeping key terms uniform and accurate. The other team members reviewed the translations. This step helps ensure that the translations sustain the nuanced meaning and context of the original responses.

Interviewing additional veterinarians was stopped when data saturation was achieved, meaning that no new data or insights emerged in the interviews (23). This was achieved after interviewing 34 veterinarians. Data saturation was measured and proved through different approaches (1) thematic saturation (repetition of patterns in the data with no new themes emerging). (2) informational redundancy (as data was collected and analyzed, redundancy or repetitiveness was noted, when new interviews or observations keep yielding same information, this might imply reaching saturation). (3) extended data collection (deliberately 4 more veterinarians were interviewed but no new data, themes, or insights emerged, confirming the reaching of saturation, those were not included in the study).

### Ethical approval

Ethical approval to conduct the study and to contact farmers was obtained from the institutional review board at our institution – IRB-REC/0/02722/3221.

With the informed consent of the interviewees, interviews were recorded and completely transcribed to avoid an analytical interpretation. Investigators assured all respondents that we aimed to map their thoughts and practices regarding antibiotic use, and not to judge them.

### Data analysis

Interview transcripts were read carefully and coded inductively by the first author, using the qualitative data analysis approach to identify emerging themes (24, 25). Coding involves labeling relevant words, phrases, or actions in the transcript and categorizing them into particular themes, with subthemes within these (26). Interview texts that were coded for the same themes were reviewed attentively and in order. The validity of the qualitative research was determined by the power and soundness of the arguments provided, the thoroughness of reporting the techniques in the study, and the re-coding and comparison of data between the researchers (27). To that aim, recoding was performed by authors 2 and 3, and the results were double-checked for uniformity by the three researchers involved in the study. Reading through the codes and focusing on how that specific subject is described helped in identifying frames that aided in theme analysis (28), such as exploring the views of the veterinarians according to the particular themes. The codes were re-read to ensure that the frames identified were accurate and that nothing was missed. During the analysis, data saturation was achieved whenever no new information was produced, and the same codes were emerging and being described in similar ways (29).

## Results

### Respondents’ characteristics

Participants’ IDs were created according to their number of responses to the interview, such as the first respondent/ first interviewee was V1, the second respondent was V2, and so on. The 34 respondents (corresponding to ~64% of the total Lebanese dairy veterinarians) provided a wide representation of the Lebanese dairy veterinarians, considering age, working experience, and governorate-working place distribution (Table 1). The results showed a range of variation among the participating veterinarians, some of whom were in their 30s with 5 to 10 years of experience, up to 50s and 60s with 25 to 40 years of experience. The gender distribution among the interviewed dairy veterinarians is skewed, with 33 out of 34 being males. This mirrors the broader context in Lebanon, where certain professions continue to exhibit gender-based preferences. This observation is supported by the database of registered dairy veterinarians in Lebanon, which indicates that out of 55 registered professionals, only 1 is female, underscoring the enduring influence of cultural and social norms on gender-specific job acceptance. Table 2 represents a summary of the mean (lowest/highest) range of each of the ages, and years of experience of the respondents, along with the gender distribution. All veterinarians possessed at least a master’s degree in veterinary sciences, some of whom also held a higher doctorate. Veterinarians with higher education (Ph.D. degree) [3 respondents] and more years of experience (more than 10) [13 respondents] were more inclined to apply best practices in herd care and farm management.

**Table 2:** Summary of age, years of experience, and gender distribution of participating dairy vets.

Distribution of respondents	Age (years)	Years of experience	Gender	
			Male	Female
Mean	42.6	15.1	97.1%	2.9%
Range (Lowest/Highest)	28/70	4/40	–	–

Moreover, many of the respondent veterinarians work in the private sector, providing their services in a specific region of Lebanon, most of which work in north Lebanon/Mount Lebanon, Bekaa, and Zahle, as a reflection of the density of dairy farms in those areas. Veterinarians in the south of Lebanon usually work with smaller farms. Five veterinarians worked as strolling veterinarians, treating dairy cattle in several farms along different governorates of Lebanon. Additionally, eight out of the 34 respondents work in the public veterinary sector under the Ministry of Agriculture.

### Veterinarians’ practices towards AMU on dairy cattle

A summary of the main indicators that emerged from the interviews is represented in Table 3. Twenty-seven out of 34 veterinarians believed that AMU in the animal industry should be limited and that AM should only be used to treat infections in animals while emphasizing that not every sick animal needs to be given AM. The clearest example of this was from veterinarians who have more than 20 years of work experience.

**Table 3:** A summary of the main indicators of responses that emerged from the interviews.

Number of respondents*	Main indicators	Themes
23	AM uses should be limited	Veterinarians’ practices towards AMU on dairy cattle
32	Diseases such as mastitis, metritis, … could be avoided by preventive measures	
34 (all)	Experienced treatment failure in their career	
21	Slaughter the animal upon successive treatment failure	*Veterinarians’ approach towards successive AM treatment failure*
29	Slaughter should not be performed before awaiting the AM withdrawal period	
12	Sick cows should be euthanized	
24	Previous knowledge and working experience	*Factors that govern veterinarians’ AM prescription decisions*
1	Always prescribes an AM with a pre-cytology test,	
29	Rarely using clinical tests diagnosis	
34 (all)	Use a broad spectra AM to target multi-resistant bacteria	
28	Prescribing AMs based on what is available at their workplace, and on the farmer’s purchasing power	
4	Prescribing unnecessary AMs, as a response to farmers’ demands, trying to fulfill their customers’ needs.	*Conditions that direct veterinarians’ use of AM as a preventive measure*
20	Overseas trade	
29	AM is considered misused when administered at underdose only	*Veterinarians’ lines towards AM misuse*
15	Both an underdose and overdose use of AM is a misuse treatment,	
28	Eager to reduce AMU on farms, as it is related to boosting the public and animal health, and reducing the AMR	*Willingness of veterinarians to reduce AMU on Farm*
28	Reducing AM means less withdrawal of milk, higher profitability for the farmer	
31	records should be attained at the farm level by the farmers	
34 (all)	AM injections are done by the farmers themselves	*Farmer-vet relationship*
34 (all)	Economic restraints have threatened their work. To reduce their costs, farmers today rely less on veterinarians	
34 (all)	Ill-farming management practices due to lack of farmers’ awareness	
34 (all)	A wide practice towards business economy profit over animal and human health care	
28	The Necessity of forcing governmental surveillance on farms	
7	Governmental surveillance should also cover vets	
34 (all)	Veterinarians affirmed that AMR is a sensitive matter that is highly prevalent in Lebanon	*Attitudes of veterinarians towards AMR and its scale threat in Lebanon*
34 (all)	AMR does not mean bacteria can resist the effect of all antibiotics	
34 (All)	The effects of AMR transmittance from animals to humans is very significant and alarming	

*Total number of respondents = 34.

*“I do only use AMs in case of inflammation, and I usually notice this by inspecting the milk, if it is like dissociated…turning to a greenish color instead of crystal white…having some coagulants and blood clots inside…then this cow carries a bacterial infection and needs an AM to be treated”* (V7, Nabatiye)

Generally, fever, respiratory infection, metritis (uterine inflammation), foot rot, and mastitis (udder inflammation) were the cases that trigger veterinarians to use AMS, with all veterinarians agreeing that mastitis is the most commonly encountered disease in dairy herds. Veterinarians described that the frequent cases of mastitis are related to a lack of health sanitation and proper management at the farm.

*“We always encounter mastitis in most cases…usually, it is about milked cows sitting on the ground, basically uncleaned and undried, before their teats are sealed, which cause bacterial infection and consequently inflammation… other cases are encountered from the farmer himself milking the cows without properly cleaning his hands, and moving between an infected case and a healthy one…infection is transmitted”* (V7, Nabatiye)

Although some veterinarians (14 respondents) reported that high-producing cows are more susceptible to mastitis, most of them (32 out of 34) agreed that such diseases could be mostly avoided by certain preventive measures, including (1) ensuring a dried cleaned area beneath cows, or (2) providing a small portion of feed immediately after milking, to push the cow to stand for a while until its teats seal, (3) Pre-dipping the teats with sanitizer, (4) post-dipping the teats with iodine solution, and (5) increasing knowledge and awareness among the farmers.

Critically important AMs such as ceftiofur, a third-generation cephalosporin with no withdrawal time for milk, neomycin, erythromycin, gentamycin, and florephenicol were among the most administered AMs by all veterinarians. Penicillin, oxytetracycline, and cephalosporin were among the most frequently prescribed highly important AMs, prescribed by 26 respondents of the dairy veterinarians.

Fifty percent of veterinarians reported side effects when AM was administered to the cattle, such as inflammation of the site of injection, skin rash, difficulty breathing, digestive disorders, diarrhea, and weakness, and most of them agreed that irregular and over-use of AM causes malfunctioning of the kidneys and the liver. All the veterinarians stated that they have experienced treatment failure in their career, which means that the animal did not improve after initial treatment. In this case, veterinarians’ behavior varies, some suggested that they might increase the dose, use the newer generation of the same AM, or just switch to an alternative AM as reported by V11.

*“After prescribing an AM based on medical field examination, if this AM did not do its expected job, I might either switch to the newer generation of the AM, as it would be more potent in its action, or substitute to another drug.”* (V11, North Lebanon, Private)

Other veterinarians reported starting treatment with a combination of AMs instead of one, claiming that this guarantees a more potent cure for the cattle. An example of this from the interview follows:

*“To avoid treatment failure, I prefer starting with a combination of two AM instead of one, such as amoxicillin and tylosine, by this I ensure a quicker recovery of the cow”* (V24, Mount-Lebanon).

Conversely, 20 veterinarians noted the necessity to respect a duration between 2 different administered AMS, since the interactions of some antibiotics might be toxic to the animal.

#### Veterinarians’ approach towards successive AM treatment failure

Veterinarians also added that in case the cow still does not respond well to the medication, they would suggest that the farmer either (1) sell the animal live, (2) slaughter and sell it as meat or (3) euthanize it (humane euthanasia); indicating that farmers usually prefer the first two choices. More than half of the interviewed veterinarians (21 respondents) affirmed that they directed the farmer to sell the animal or slaughter it as meat once the cow was successively treated but uncured.

*“In case the cow was not cured after alternative treatment trials, I suggest slaughtering the animal and selling it for meat, but after properly treating it and waiting for the respective AM withdrawal period”* (V20, Strolling Veterinarian)

*“I recommend directing the un-recovered animal specifically to industries for processing as canned meat, since under elevated temperature of sterilization and vacuum that canning process go through, the disease would be killed as well as the AM”* (V3, Baalbeck-El Hermel)

Almost all veterinarians (30 respondents) agreed that farmers should not slaughter or sell the cow before waiting for the withdrawal period of the AM. However, it was clear from the veterinarians’ responses that farmers would go for the option of culling and selling the cow if it is chronically sick and unproductive. Veterinarians highlighted that most of the farmers do sell their cows for meat production while being under antibiotic treatment or suffering from inflammation. This practice of street market sales without inspection is illegal.

*“I do notice a lot of cows at the slaughterhouse with many bruises in their body due to several needle injections…and it is simply sold like that!”* (V2, Nabatiye).

Few veterinarians (12 out of 34) insisted that chronically sick cows should be euthanized, rather than sold or slaughtered for meat. However, veterinarians complained that farmers do not rely on their advice and that they prefer to take more profitable actions on their own.

*“Usually, when we give up on the possibility of a cow recovering from its illness, we suggest to the farmer to euthanize it and bury it, we call it ‘merciful death” However, I do know that the farmer does not listen to my recommendations and that instead they sell it at a lower price in poorer areas of the country…according to the farmer, lower price is better than nothing, regardless of the serious health concerns of the issue”* (V1, Mount-Lebanon)

Interviewer: *“Why have not you reported this to the governmental authorities?”*

Respondent: “*We already did, they do know about it, but they just do not follow it up properly*…” (V1, Mount Lebanon)

#### Factors that govern veterinarians’ AM prescription decisions

To understand the reasons behind AM treatment failure, veterinarians were asked about what governs their decision regarding specific antibiotic prescriptions. Twenty-nine veterinarians argue that their specific choice is built on several factors, including previous knowledge and working experience, the respective organs affected, and the potential pathogen involved. Few veterinarians (9 respondents) affirmed to develop a specific strategy for the use of first-line therapy for common conditions, always using the same sort of things for the same type of problems.

*“Along my years of work experience, I have developed static tactics, perceiving immediately the AM that would be most efficacious against the most likely microorganism involved, such as always prescribing oxytetracycline and penicillin for mastitis and cephalosporin for laminitis…”* (V5, Bekaa-Zahle)

Other veterinarians consider the animal’s medical history and approach farmers from various angles to assess if any information is withheld before prescribing specific antibiotics. To follow up on this topic, veterinarians were asked whether they rely also on a diagnostic confirmation test before prescribing AM. Different behavioral practices were shown, only one veterinarian highlighted that he never prescribes an AM without a pre-cytology test, while 11 of them rely on farm physical examination, the majority of veterinarians assist their previous experience along with the farmer’s description of the case as accountable for making up their treatment decision. Those cases are demonstrated by the following examples:

Interviewer: *“So basically you made up your decision based on previous experience and physical examination, or you go for a diagnosis test before treatment for confirmation?”*

Respondent 1: *“Let me tell you that I have got my specific laboratory within my clinic, and I do not prescribe any AM without a cytology gram staining test for microscopic examination, regardless of the nagging of the farmers… now all my customers know that this is my strategy and this how I follow up, point”* (V2, Nabatiye)

Respondent 2: *“I base my decision on previous knowledge, and my experience on treating similar cases…and 60% on how the farmer customer describes the case”* (V8, Mount Lebanon)

Respondent 3: *“I examine the cow by visual inspection, using the stethoscope and the thermometer, I can tell what is the disease or the suspected case to know what medicine to prescribe…it would be better of course to take a biopsy for confirmation…but…sincerely it is very money consuming in our hospitals and laboratories compared to outside in Europe and USA where it is almost done for free!”* (V18, Nabatiye)

Only 5 veterinarians mentioned conducting a diagnosis test before prescribing an AM and that this was not done on a routine basis. The majority of veterinarians (29 out of 34) reported rarely using clinical tests diagnosis, basing their decision solely on their working experience. Similar to V18, almost all veterinarians identified various barriers that prevented them from applying an AM sensitivity test, starting with the unavailability of established laboratories along the regions of Lebanon for such cases. Emphasizing that if laboratories were present, there would still be a time delay in the results of the diagnostic test which most veterinarians complained about, explaining that farmers prefer short-duration consultation and rapid monitoring examinations. Above all, veterinarians mentioned that farmers refrained from the AM sensitivity test due to their unwillingness and inability to pay for it. To report on this issue, some of the veterinarians’ complaints that retarded their access to clinical diagnosis tests are highlighted in the following examples:

*“There is one laboratory in Mount Lebanon and another in Bekaa, but I am living in the North, taking samples there would take around 3 to 4 hours by car, it is tiring, time-consuming and ineffective”* (V11, North Lebanon)

*“I would love to take samples for confirmation before AM prescription, this is how it should be, but we are facing a big problem concerning the lack of laboratories in Lebanon, especially in Akkar governorate”* (V20, Strolling Veterinarian)

Few veterinarians (11 respondents) also added that laboratories in Lebanon, if available, lack proper assistance and accuracy in results, and therefore cannot be trusted. On the contrary, only one public veterinarian mentioned that there is a specialized laboratory at the Ministry of Agriculture and that free samples are allowed to be collected and analyzed there.

Almost all veterinarians (30 respondents) determined that they go for a broad-spectrum AM. However, some veterinarians fluctuate in their decision regarding the spectrum of AM prescribed, arguing that they use both broad spectra AM and narrow spectrum AM according to the case.

*“I might use a broad-spectrum antibiotic if I have some doubts towards the infection case, and I will go for a narrow spectrum AM if I am pretty sure of the situation”* (V16, Strolling Veterinarian)

All veterinarians also reported that they use broad-spectrum antibiotics, targeting multi-resistant bacteria, claiming that this is more assured and effective in the treatment of infections and diseases. As for the mechanism of AM delivery, veterinarians stated that their most frequent route of AM administration is local and systematic or via intravascular injection, though few affirmed that they follow the oral prescription route via water or feed.

Veterinarians reported as well that they might seek guidance with the AM choice from their colleagues, books, or drug companies. The majority of Veterinarians confirmed prescribing AMs based on what is available at their workplace, seeking this as a more facile opportunity.

*“Usually, I rely on what formulations are available at my workplace, it is much easier to prescribe from them and promote immediately to the farmer”* (V15, Bekaa-Zahle)

Twenty-eight out of 34 veterinarian respondents had similar views with V15, and some also highlighted that they possess different quality standards of the same AMs at the workplace, roughly of which are manufactured in European countries with purer molecules, considered as first-degree medicine, while others are made in the Middle Eastern with less efficacy and hence lower prices (second-degree medicine). Veterinarians acknowledge that they direct the cost of the therapy and the explicit quality of AM according to the farmer’s purchasing abilities whether they are from a considered-rich neighborhood (involving usually Beirut, Bekaa, and Mount Lebanon) or from a poorer one (Nabatiye, Akkar, North and South Lebanon).

*“Of course, the social status of the farmer dictates our AM prescription approach. In my clinic, I have high-quality product standards, forex, and drugs that are made in Holland, considered first grade, and sold at 12 – 13$, while 70 to 60% of the other items are made in Syria, Egypt, or Sri Lanka at a lower quality that does not comply well within the standards and only sold at 1$…however, the bad economic situation has made the second choice a more feasible option for the farmer.”* (V1, Mount Lebanon)

Through this quote, veterinarian 1 explicitly demonstrated the conditions of service treatment in Lebanon, reflecting that generally, the cheapest drug was often the respective choice of most farmers, especially amid the tremendous economic crisis Lebanon has been facing since 2019.

#### Conditions that direct veterinarians’ use of AM as a preventive measure

Moreover, some veterinarians admitted that they use AM as a preventive action and to promote growth in cattle. Very few (only 4) veterinarians mentioned that they follow this approach, prescribing unnecessary AMs, as a response to farmers’ demands, trying to fulfill their customers’ needs.

*“I do sometimes prescribe AM as a preventive measure for the whole herd… this is what my customer enquires…and I need to fulfill his desire…”* (V5, Bekaa-Zahle)

Others highlighted that metaphylactic approaches are a must on farms, claiming that once a cow got infected, the other cows in the herd would be affected as well, via various common routes they share in a small-land farm. A clear example from the interview follows:

*“Let me tell you something…you are having all the cows feeding on fodder from the same place…drinking water from the same place…living in a confined area…so, if one cow got infected, then 99% that this bacteria would be transmitted to the other cows as well, what I do is that I just provide the AM to the whole herd immediately as a metaphylactic measurement…”* (V12, North and South Lebanon)

Additionally, veterinarians stated that the majority of the available beef herd in Lebanon is from an imported origin, as Lebanese farmers prefer European and Brazilian cows over national ones, stating that they are more productive. On such occasions, some veterinarians are obliged to use prophylactic measures during herd export, as cows are susceptible to various diseases during overseas transport. According to veterinarians, overseas cattle trade is a critical situation that is accompanied by disease transmission from the country of origin to the destination. Some veterinarians reported that most of the time, diseases are acquired along the journey due to the several fluctuations of weather and the tightened space available for the cattle, which prominently oblige the use of AMS.

“*For me, delivering AM treatment to the dairy herd during overseas trading is a must… the tight occupation, weather fluctuations, and the overseas sickness hit up the herd always…and as such, I go for the prophylactic treatment”* (V6, Strolling Veterinarian)

#### Veterinarians’ attitudes towards AM misuse

Most veterinarians (29 respondents) determined that AM is considered misused when administered at underdose only, while others referred that both an underdose and overdose use of AM is a misuse treatment, signifying different perspectives of veterinarians that are reflected in their practices.

*“I always prescribe AM at a higher dose, approximately a dose and a half, I found this more practically rewarding, as I am attacking the bacteria with a higher power, and cows consequently recover quicker”* (V11, North Lebanon)

In this quote, the veterinarian describes how he instantly prefers to go for a dosage above the label recommendation to regain a faster recovery of the cow. Besides, very few veterinarians (6 respondents) followed a more static strategy, emphasizing that an AM dosage should be administered according to the weight of the cow, going neither above nor beyond this specific margin. This is demonstrated by the following example:

*“I assign the dose of AM according to the weight of the cow, for ex, 1 ml drug for every 10 kg weight, if a cow is 50 kgs, then it is a 5 ml dose of AM. Now if this was administered below the recommended level, the remaining un-killed bacteria would create immunity against the antibiotic…whereas an overdose administration means the treated bacteria get acquainted with the highest dose, next time you got to re-increase the dose… and the animal might not endure it…this is life-threatening as well!”* (V2, Nabatiye)

#### The willingness of veterinarians to reduce AMU on farm

On another side, most veterinarians (28 out of 34) were willing to reduce AMU on farms by promoting preventive measures (health sanitation, vaccination protocols), prioritizing accurate diagnosis, and monitoring animal health. Veterinarians expressed that this would reflect many advantages, including boosting public and animal health, reducing the AMR, lowering the withholding quantity of milk and meat, and improving the image of the dairy industry.

On the other side, most veterinarians (25 respondents) were still worried that the lack of farmer’s knowledge, proper management, and adherence to rules could retard the applicability of this situation. Some veterinarians reported that improving the animal’s health through regular vitamin administration, vaccination, along proper herd management, could significantly reduce the need for antimicrobial treatments.

It was clear from the interviews that there is nonobservance of withdrawal periods of administered AMs before cows are milked, sold, or consumed among most farmers. Most veterinarians (29 respondents) disregard this out of their responsibility.

*“After I provide my examination, I do not contact the farmer to make sure whether he committed well to the medical plan or not, it relies on the farmer’s consciousness and awareness”* (V33, Baalbeck-Hermel – public)

This shows that according to veterinarians, their interference part ends up after prescription, and that following the withdrawal period of AMS is not their responsibility. Moreover, when asked whether they keep medical records for treatment cases of dairy cattle on farms, almost all veterinarians claimed that such records should be attained at the farm level by the farmers and that it is out of their responsibility.

*“I am not the one who should be keeping the medical records, such documents should be kept by the farmer for his dairy cattle at the respective farm…had I been treating a cat or a dog at my veterinary clinic, I would then keep a record”* (V24, Mount Lebanon)

Considering that in the study of Dankar et al. (5), farmers in Lebanon reported an absence of record keeping on farms for cattle medical cases.

### Farmer-veterinarian relationship

Antimicrobials administered by veterinarians likely contribute to only a very limited extent to the overall antimicrobial consumption on dairy farms. All veterinarians explicitly stated that most of the treatments and AM injections are done by the farmers themselves. Farmers were classified accordingly into 3 groups: (1) those who prescribe AM and administer it by themselves, (2) those who take the suggestion of the veterinarian but administer it improperly by themselves, and (3) those who rely merely on veterinarians to treat their cattle. The behavior of the first 2 groups is widespread among the Lebanese dairy farmers, principally amid the economic constraints the country is passing through while the last group is constrained only to large dairy farms and is limited in Lebanon.

*“Amid the economic collapse Lebanon is facing, farmers are more willing to take their actions by themselves and ignore the bill of a veterinarian…I can specifically tell you that the rate of our work lately has slowed down by 90%”* (V19, Bekaa-Zahle)

As reflected by Interviewee 19, the economic restraints have threatened their work. To reduce the costs of paying for veterinarians, farmers in Lebanon today rely less on veterinarians and prefer to make most of the decisions freely on their own. Moreover, veterinarians are aware that antibiotics can be obtained from different sources in Lebanon: a veterinarian, a feed store, over-the-counter supermarkets, or directly from drug distributors. According to veterinarians, this is illegal, and farmers should be strictly directed to buy the antibiotic only from the pharmacy and upon a veterinarian’s prescription. This is elaborated by V2 in the quote below:

*“Under authority regulations, companies importing drugs to Lebanon should only distribute it to pharmacies or veterinarians, and farmers must only be allowed to buy the respective medicine following a confirmed veterinarian prescription”* (V2, Nabatiye)

Most veterinarians (27 respondents) also highlighted how the accumulation of AMs at farms facilitates improper treatments, ignorance, and irresponsible acts from the farmers. Examples come as follows:

*“I do know that most farmers keep at their farms those plastic bottles, containing random mixes of AMs…sometimes over 4 to 5 simultaneously! They also store bottles in the sun, and they just use them whenever they want to…really ignorance and a whole mess!”* (V18, Nabatiye)

*“I do know a lot of farmers that make up their minds and use the AM that they are most familiar with regardless of whether the veterinarian is convinced towards this choice or not”* (V6, Strolling Veterinarian)

Veterinarians enlightened that the lack of education and awareness among most farmers is very alarming, consequently leading to ill-farming management practices and the spreading of different diseases.

*“You know, for instance, a cow infected with foot and mouth disease, which has a high tendency to spread among the herd…is not isolated, and simply kept among the cattle, you end up with the whole herd being infected…there is no culture of animal isolation, adding to this, the lack of capabilities of the farmer and the absence of support from the government*” (V1, Mount Lebanon)

*“You have mastitis, the most commonly acquired disease among dairy cattle, it got more profound and harder to treat due to the absence of awareness between the dairy farmers, and their delay in contacting a veterinarian and immediately starting the treatment!”* (V6, Strolling Veterinarian)

All veterinarians also mentioned that in addition to the lack of awareness among most dairy farmers, there is a wide practice towards business economy profit over animal and human health care, specifically amid the current economic crisis in Lebanon.

*“Mainly farmers do not care about people, what they worry about is whether their milk processing procedure is going to work or not…I do know that a large group of Lebanese dairy farmers use the milk during the withdrawal period to formulate different kinds of cheeses, following the concept that if this milk cannot form yogurt, it will perform well in other options…primarily caring to avoiding economic loss, especially amidst those financial constraints”* (V2, Nabatiye)

Some veterinarians felt despaired about the random and unorganized administration of AMU at Lebanese dairy farms, as well as the lack of herd sanitation and management, to the extent that they could not trust a meat product from a Lebanese origin, following their consumption solely from an imported meat:

*“Since 5 years ago, I have only bought imported meat from European countries from supermarkets…I never allow locally produced meat to enter my home…I need to protect myself and my family, especially after all that I have witnessed and still witnessing daily among several dairy farms in Lebanon…simply, knowledge and awareness are null…herd treatment, follow-up, and management are big chaos!”* (V10, Strolling Veterinarian)

Veterinarians urgently called to raise awareness and knowledge among Lebanese dairy farmers, stressing their responsibility to educate farmers and improve farm management to prevent diseases and reduce antimicrobial use on cattle. In this trend, all private veterinarians directed a message to the employers at the Ministry of Agriculture, requiring that proper management should be achieved with stronger surveillance and inspection even at the level of the dairy industry, believing that once we start there, we would restrict and shape the AMU behavior at the level of the dairy farm.

*“We should impose sturdier inspection on farm management…distribute free vaccination…but what I think is most important is to strictly investigate cheese production at dairy industries, forcing them to stop collecting milk with Ab residues from the market…by this, farmers would be more obliged to be persistent in their AM practices…”* (V6, Strolling Veterinarian)

A few veterinarians (7 respondents) also mentioned that strong surveillance should involve the veterinarians and not only the farmers making sure that veterinarians are taking the right actions and fulfilling their jobs. Furthermore, all veterinarians affirmed that the severe economic situation in Lebanon and the absence of any governmental support for the agricultural sector, has made the conditions worse and harder on the burden of the farmer, where prices had increased almost 25 times, revealing that what the farmer could get and manage easily before the economic crisis is now a real hurdle to access.

*“The dollar has increased from 1500 LL to 30,000 LL, a huge leap! … all prices here are in dollars, the fodder, the sanitation, the medicine… those has marked as huge stressors on the choices of the farmer, shifting them to use the cheapest not the best…”* (V23, Akkar)

Almost all veterinarians (32 respondents) asserted that farmers could not withstand all this load and that the government should not only implement surveillance and force regulations, but also play its role in supporting the farming sector by providing free vaccines, free pasture, or milking systems for small farms to serve as a tool for proper management.

*“Despite raising awareness and carrying out training sessions, the farmer will not be able to make the right choices had he not got the economic feasibility”* (V26, Mount Lebanon)

However some veterinarians acknowledged that larger Lebanese farms (generally having 100 lactating cows and above) take better care of their cows, their sanitation, and their farmland, compared to average-sized farms (owning 15 to 40 lactating cows). Veterinarians reported that each large farm (although very limited) has a full-time veterinarian. A description of the situation in the Lebanese large farms is reflected in the following quote:

*“I am a fixed veterinarian on one of the large farms in Lebanon, and I can assure you that we apply everything strictly and we take into consideration all details, from the free available space per cow in the herd to the sanitation and material of the wall that would prevent insect infestation, changing workers’ clothes, boots and gloves daily…not allowing visitors to enter without wearing the specific uniforms…and of course daily routine inspections of the fodder, the health status and sanitation of each cow…”*(V14, Bekaa-Zahle)

### Attitudes of veterinarians towards AMR and its scale threat in Lebanon

All veterinarians affirmed that AMR is a sensitive matter that is highly prevalent in Lebanon and that serious actions should be taken immediately. According to veterinarians, AMR is the ability of a bacteria to survive exposure to an AM that was previously an effective agent. In other words, AMR implies the non-response of the cow to curing from microbial infection, and consequently treatment failure. Veterinarians summarized the reasons that facilitate AMR as the following: (1) chaotic use of AMs by the owner without referring to a veterinarian, (2) using incorrect combination of an AM to a disease case, (3) ill-prediction of weight to AM dosage ratio, (4) lack of commitment regarding the duration of AM course, (5) muscular administration of AM instead of intravascular, and (6) storing AM open and exposing it to the sun. One of the veterinarians provided an example where the farmer does not comply with the prescribed plan and prefers to act on his own, due to lack of awareness.

*“An AM treatment period is supposed to be for 5 days, yet the farmer does not complete the cycle and stops after the third day, assuming that the cow has recovered and that no more visual clinical signs of the disease are observed…however, the farmer was unaware that the bacteria was still present at small amount, it then gets stronger and thus develops resistance against the AM”* (V11, North Lebanon)

All veterinarians made it clear that AMR does not mean bacteria can resist the effect of all antibiotics, it is only against the misused AMs that were administered previously. Moreover, some veterinarians spotted that AMR could emerge through spontaneous changes in the bacterial genes due to mutations, even at a relatively low percentage, and that infections with multi-resistant bacteria are difficult to treat since the alternative for treatments are strongly limited. Concerning the seriousness of AMR amongst animals, most veterinarians say that AMR can even spread from one animal to another and that this should be taken into consideration by isolating the treated case animal away from the herd.

*“I believe that AMR could transmit from an animal to another via the fecal, the environment, the breath, the manure…not genetically but through natural factors as I said…so it is much better to treat an infected case animal in isolation”* (V9, Strolling Veterinarian)

In this quote, the veterinarian reflected how AMR can be transferred to the herd via shared location and environment which necessitates the quarantine of the infected animal away from the herd. Besides, veterinarian 6 had another point of view that AMR is transmitted genetically between the animals rather than due to environmental factors.

*“of course, AMR could be transmitted from one animal to another, such as from the cow to its calf and many next generations”* (V6, Strolling Veterinarian).

Though few veterinarians (8 respondents) believed it is illogical for AMR to spread from one animal to another unless they all consumed the same medication.

When relating the problem of AMR in animals to that of humans, very few (around 6 interviewees) related that AMR in humans depends only on the incorrect use of AM for humans and not for animals. Nevertheless, the majority of veterinarians emphasized that the AM residues and the pathogens in cows can undeniably pass to humans through the consumption of contaminated milk and meat, thus leading people to develop AMR cases similar to that found in animals, this is explicitly stated by the following quoted example:

*“Selling the milk with AM residues would seriously affect the customer’s health, once the person got infected with a similar disease, the same antibiotic that was provided to the cow and that was residing in the dairy products he/she consumed will not be effective on his/her body, since the person would already have developed resistance against this AM.”* (V18, Nabatiye)

Almost all veterinarians (28 respondents) added that the effects of AMR transmittance from animals to humans are very significant, regarding widespread cases of noncompliance to microbial infection treatment, an added treatment expense, and a longer duration of illness and therapy. Moreover, Interviewee 16 reported the identification of severe AMR cases in cancer patients, directly associating them with the inappropriate administration of antibiotics to livestock.

*“Many AM residues are a real harm to human health, for instance, dioxin residues in the human body might trigger cancer diseases in the future.”* (V16, Strolling Veterinarian)

Some veterinarians reflected that the cases of AMR are increasing and that this could also be attributed to globalization, the rapid worldwide transport and transfer among herds which have increased the incidence of disease occurrence and bacterial infection, and in turn amplified the level of AM prescriptions and consequently the rates of AMR. For this, few veterinarians roughly followed certain strategies to constrain the AMR and its threat in society. Some veterinarians tried to disregard AM as first-line therapy and to focus on preventive health care measures instead, while others suggested alternating between different kinds of medications now and then to sustain the occurrence of AMR. Some of the strategies followed by veterinarians to reduce the incidence of AMR in livestock are exemplified in the following quotes:

*“I do not like starting immediately with an AM, I prefer to provide first anti stressors to give the animal body a chance to develop its defense mechanism, its “Immunity”* (V9, Strolling Veterinarian)

*“Every 5 years I change the AM to reduce the AMR incidences”* (V12, North and South Lebanon)

## Discussion

To the best of our knowledge, this is the first study that provides insights into the attitude of dairy veterinarians towards AMU and AMR issues in Lebanon. This study is complementary to a study by Dankar et al. (9) involving the behaviors of dairy farmers towards AMU in Lebanon. This study investigates from veterinarians’ perspectives what influences their decisions related to prescribing AMs, their relationship with farmers, their attitude to the Lebanese farming management, and to the AMR issue. It is of high importance to understand the veterinarians’ practices and approaches as they are seen as the potential animal health advisors and the main rulers for implementing farm management planning and reducing AMU (30).

Compared with Dankar et al. (9), the response rate in dairy farms was 18 out of 100, while in this study, it was 34 out of 50, with a greater percentage of responses, and thus a better presentation of the scenario from veterinarians strolling several dairy farms in different regions of Lebanon.

Veterinarians with a higher education degree (Ph.D. degree) and extended experience (more than 10 years) were more willing to implement best practices for herd treatment and farm management. Their decisions were also more trusted by farmers. Likewise, Higgins et al. (31) showed that veterinarians with longer years of experience were more knowledgeable, confident, and trusted in their prescribing decisions.

In this study, veterinarians disclosed that owners of different types of farms employ distinct AMU strategies: those with larger farms prioritize long-term gains, while smaller farm owners focus on immediate, short-term outcomes. Similarly, Heederik et al. (32) reported great variation in AMU between farms in the Netherlands, indicating that there is still room for further improvements.

The decision-making process involved in the selection of AM in farm practices was complex. The demographic region of the farmer or the willingness and the ability of farm owners to pay governs the chosen strategy of medication for veterinarians when selecting the type of formulation, and therefore of substance. The conflict between best practices and AM selection by veterinarians was observed. Similarly, Mateus et al. (22) observed that the cost of therapy was found to be highly influential in the selection process of AM specifically in areas with low, mixed socioeconomic status. The study revealed obstacles in the laboratory diagnosis process. These obstacles included farmers’ inability to pay for tests and the lack of available laboratories across the country, leading veterinarians to make arbitrary or field-based decisions regarding suspected diseases and corresponding antimicrobial treatments, a paradigm that is prevalent in impoverished countries (13, 33). In developed countries like the UK and Scotland, veterinarians faced a shared challenge when it came to using lab tests because it demands significant time for results and treatment initiation, whereas farmers seek faster remedies (34).

While one veterinarian in the public sector noted that free laboratory services are available through the ministry, this was limited to the Nabatiye region. Therefore, the government should allocate more funds to establish additional laboratories in various regions of the country. This initiative should be accompanied by a call for enhanced surveillance to systematically gather information on circulating pathogens and the evolving bacterial resistance profiles.

A high incidence of treatment failure was reported in this study, which might be due to the wrong treatment, or incorrect diagnosis, as again laboratory tests were seldom used to confirm prescription decisions and the treatment therapy. Another reason could be related to the widespread of AMR among the herd dated to a limited number of drugs used on farms and to farmers and veterinarians accustomed to using the same products repeatedly (35). The antibiotics available in the Lebanese market today are very limited, especially amid the economic crisis, which is similar in the case of medications, laboratory equipment, and vaccines, among others (36, 37).

Alarmingly, some veterinarians have reported the use of third and fourth-generation cephalosporins, which are considered critically important antimicrobials by the WHO (38). Veterinarians’ replies were very serious regarding the situation of the AMR in Lebanon, most of them were aware of its escalating threat in both humans and animals, yet the actions to restrict the AMR spread are still ineffective.

This study revealed that although veterinarians were aware of the AMR threat, they did not seem to understand it properly, as evidenced by some of their acts, as it was believed that heat and vacuum remove antibiotics, however, research emphasizes that antibiotics are not degraded under heat and physical treatments (39, 40). Other knowledge gaps emerged from the veterinarian interviews reporting that muscular injection is more likely to promote AMR than intravascular injection, and that changing the antibiotic drug every 5 years is likely to have a significant effect on reducing AMR. Yet, it is the optimal treatment that must be adapted to the resistance profile of the infecting agent in each case and if this is not possible at least some updated information of the resistance profiles of circulating pathogens should be used. Recent studies highlight the concerns of medical doctors regarding the increasing cases of pathogenic bacteria that are resistant to antibiotics and their spread in the human population. The overuse and misuse of antibiotics in the veterinary, livestock, agriculture and medical (hospital and community) sectors were reported as the main reasons that have led to increasing levels of antimicrobial resistance (41, 42). The studies have identified that the problem of AMR has intensified due to the increasingly uncontrollable way of inappropriate use of antibiotics (43–45). Therefore, veterinarians should adhere to their role in following precise and confirmed antibiotic prescribing decisions (31).

Many research papers reported that reducing AMU in livestock is an important step towards reducing AMR levels in farm animals and consequently among humans (46, 47). The significance of farmers’ compliance in implementing preventative measures on farms to lower illnesses and AMU in livestock animals, as well as the crucial role of veterinarians as consultants for farmers in this regard, have been emphasized (48). Our study showed that some veterinarians worked effectively towards implementing judicious AMU on farms, expressing that reducing AMU on farm is important but they find it extremely hard to achieve due to the lack of implementation of governmental rules and regulations. Rules and regulations are essential pillars to improve human behavior, in addition to other factors such as education, social pressure, and economic support (49). All are tools within the RESET Mindset Model, lacking in the majority of developing countries (16, 50–52).

The management of a veterinary practice as a business was discussed by some respondents. Our study showed that some veterinarians use AM orally via water or feed and as a preventive measure, which justifies the irresponsible acts of some veterinarians, their lack of confidence and total compliance to farmers’ needs especially amid the economic constraints in Lebanon and their fear to lose their jobs. Likely, veterinarians’ practices in small farms at United Kingdom linked their decisions regarding AM prescription and administration to satisfy client’s expectations despite that the prescribed AM might not be the best choice, resulting in excessive AMU (22). Gibbons et al. (53), also mentioned that veterinarians were more likely to prescribe if they sensed the farmer expected them to prescribe AMs. It is important to emphasize that such acts are not only due to the compliance of the veterinarian to the farmer’s demands but also as a result of a lack of proper scientific knowledge and awareness for few veterinarians, who believed that an overdose and oral prescription is a suitable approach for treatment and cattle recovery. Moreover, a very serious and unconscious act was condoned by some veterinarians, as they advised farmers to sell their sick live cows or to direct them to slaughtering for meat production. Such uses can result in a number of problems, including the emergence of antibiotic-resistant bacteria, human and animal illness, and public health threat (35). Accordingly, raising awareness and good practices should be inclusive to veterinarians and not only to farmers. In this context, WOAH established a plan to control the risk of AMR in animals and population, which include (1) well-trained veterinarians and veterinary paraprofessionals are at the forefront of national and regional efforts, (2) ensure engagement between veterinary services and farmers, (3) provide assistance and leadership to governments as they implement national action plans and policies, and (4) overall promote the “One Health” approach which issues the interconnectedness of the health of humans, animals, plants and ecosystems (8).

Many studies and organizations highlighted the compulsion of record keeping for proper AMU and consequently to reduce the spread of AMR (54). A record-keeping should not only include the reason for treatment and occurrence of a disease, but also the treatment duration, the interval between successive administrations, and the dose of the used antimicrobial (54). For instance, Fertner et al. (55) highlighted that every drug for veterinary use must be registered in the Danish national database VetStat, which holds detailed information on each purchase of the drug such as the date, prescribing veterinarian, receiving herd ID, species, age group, and clinical indication. Unfortunately, in this study, those data were unavailable from both the dairy farmer and the dairy veterinarian side, implying (1) the weak dairy farmer-veterinarian relationship, (2) the lack of awareness among dairy farmers, (3) the absence of veterinarians’ role towards implementing their expertise and educating dairy farmers, and the (4) lack of enforcement of governmental rules and regulations.

The observation in this study shows that the majority of AM were used systematically in accordance with the findings of the Canadian study (56), but in contrast to other studies where the majority of AM were infused at an intramammary level (57, 58). A reduction of systemically used antimicrobials could be reached by enhancing general animal health care. Less systemic critically essential (long-acting) wide spectrum antimicrobials would be then required (54). This change will need a significant attitude shift from both dairy producers and veterinarians. Some veterinarians showed unwillingness to reduce AMU on farm, fearing the treatment failure of cows and an extra work burden on the farmer’s shoulders. However, a study conducted by Speksnijder et al. (48) showed that reducing AMU gradually on farm did not result in any adverse effects on animal health and productivity, and that this was achieved by adjusting management practices in a team effort among veterinarians and farmers.

Most of the interview responses revealed the lack of communication and trust between the dairy veterinarians and the farmers, some veterinarians have declared that nowadays they are working much less than three years ago which confirms that farmers are more relying on their own choices and departing totally from the veterinarian’s paid service. Farmers might only consult a veterinarian when they feel that their animal has reached a clinical stage that can no longer be ignored (35).

The fact that farmers take actions on their own, regarding the access to AM via counterfeit supermarket without medical prescription, administering AM by themselves without veterinary consultation, not complying to the AM course, and selling dairy products and meat without waiting for AM withdrawal period is alarming. Unfortunately, this was coupled with the absence of regular herd visits from a veterinarian. It was clear that some veterinarians revealed the lack of proper monitoring and advice sharing to farmers, by showing no interest in continuing to examine the treated case, nor checking whether the farmer had completed the AM course effectively, claiming that farmers would always do what they have on their mind regardless of their advice or intervention. However, it is essential to highlight that veterinarian’s behavior is of high importance with respect to influencing a farmers’ behavior (31, 59). Sustaining reckless acts by veterinarians could generate irresponsible and inappropriate actions regarding herd management and AMU by dairy farmers, producing a closed loop of irregular performance, knowing that the role of veterinarians relies on encouraging farmers the implement positive actions in a continuous cycle of improvements (60).

Attempting to do this is challenging, and it frequently fails. According to Scherpenzeel et al. (61) a strong farmer-veterinarian relationship is necessary to achieve judicious of AMU. Together, they should issue a herd health plan and a herd treatment plan that is based on the actual herd situation. The herd health plan should be farm-specific, define the main points of disease prevention, and include appropriate protocols for diseases that are treated on-farm without veterinary oversight (61).

We recommend implementing guidelines as a reminder of best practices not only for farmers but also for veterinarians. In a similar attempt, Mateus et al. (22) found that action should be taken to raise awareness of veterinarians via targeted training in the workplace through effective meetings, seminars, or mentoring schemes to promote responsible AMU and raise AMR awareness (22).

Several limitations apply to this study. Recall bias among veterinarians may have led to less reliable information in the reporting of drug treatments. It is also possible that social desirability could have influenced some of the answers regarding proper drug prescription and management on farms. However, even if those veterinarians represented the best-case scenario, we were able to deepen the understanding of the patterns and indications for antibiotic uses, farmer-veterinarian relationship, and AMR status that may reflect some overall trends in low-income countries with the dairy and veterinary sectors.

## Conclusion

The findings of this study were innovative and supported the feasibility of qualitative approaches in animal farming research in developing countries, which aids in enhancing policy formulation and effectively executing current standards for responsible AMU. The findings are concerning, and they must be taken seriously when driving national and international policies. Farmers and veterinarians have a shared responsibility in accounting for prudent uses of antimicrobials on dairy farms to limit the spread of antimicrobial resistance. This should be supported by the Ministry of Agriculture, and the Veterinary Association Committee in Lebanon, by launching educational programs to encourage judicious use of antimicrobials in livestock for farmers and veterinarians. Immediate interventions that should be considered in Lebanon include, banning the use of critically important antibiotics in livestock and prohibiting the purchase of AM drugs without a veterinary prescription. Additionally, the possible conflict of interest between promoting ethical antimicrobial prescription and losing clients could be overcome by improving communication between veterinarians and farmers, unifying their approach.

## Data availability statement

The raw data supporting the conclusions of this article will be made available by the authors, without undue reservation.

## Ethics statement

Ethical approval to conduct the study and to contact farmers was obtained from the institutional review board at our institution – IRB-REC/0/02722/3221. With the informed consent of the interviewees, interviews were recorded and completely transcribed to avoid an analytical interpretation. Investigators assured all respondents that we aimed to map their thoughts and practices regarding antibiotic use, and not to judge them.

## Author contributions

ID: Data curation, Investigation, Methodology, Writing – original draft, Writing – review & editing. HH: Conceptualization, Project administration, Supervision, Writing – original draft, Writing – review & editing. MS: Conceptualization, Project administration, Supervision, Writing – original draft, Writing – review & editing.
